# Machine learning based efficient prediction of positive cases of waterborne diseases

**DOI:** 10.1186/s12911-022-02092-1

**Published:** 2023-01-18

**Authors:** Mushtaq Hussain, Mehmet Akif Cifci, Tayyaba Sehar, Said Nabi, Omar Cheikhrouhou, Hasaan Maqsood, Muhammad Ibrahim, Fida Mohammad

**Affiliations:** 1 Department of Computer Science and Information Technology, Virtual University of Pakistan, Lahore, Pakistan; 2 Department of Computer Engineering, Bandirma Onyedi Eylul University, Balıkesir, Turkey; 3 Informatics, Klaipeda State University of Applied Sciences, 91274 Klaipeda, Lithuania; 4 CES Lab, National School of Engineers of Sfax, University of Sfax, 3038 Sfax, Tunisia; 5 Department of Information Technology, The University of Haripur, Haripur, Pakistan; 6 Department of Computer Engineering, Jeju National University, Jeju-si, South Korea

**Keywords:** Machine learning, Patient information, Malaria, Typhoid, Waterborne disease

## Abstract

**Background:**

Water quality has been compromised and endangered by different contaminants due to Pakistan’s rapid population development, which has resulted in a dramatic rise in waterborne infections and afflicted many regions of Pakistan. Because of this, modeling and predicting waterborne diseases has become a hot topic for researchers and is very important for controlling waterborne disease pollution.

**Methods:**

In our study, first, we collected typhoid and malaria patient data for the years 2017–2020 from Ayub Medical Hospital. The collected data set has seven important input features. In the current study, different ML models were first trained and tested on the current study dataset using the tenfold cross-validation method. Second, we investigated the importance of input features in waterborne disease-positive case detection. The experiment results showed that Random Forest correctly predicted malaria-positive cases 60% of the time and typhoid-positive cases 77% of the time, which is better than other machine-learning models. In this research, we have also investigated the input features that are more important in the prediction and will help analyze positive cases of waterborne disease. The random forest feature selection technique has been used, and experimental results have shown that age, history, and test results play an important role in predicting waterborne disease-positive cases. In the end, we concluded that this interesting study could help health departments in different areas reduce the number of people who get sick from the water.

## Introduction

Pathogens, biotoxins, and pollutants in contaminated water cause waterborne diseases like schistosomiasis, cholera, and other stomach problems [1]. The transmission or spread of these pathogenic microbes happens due to bathing, drinking contaminated water, washing, and eating food exposed to contaminated water, among other things [2]. Vomiting and diarrhea are the commonest symptoms of waterborne diseases; other symptoms include skin, eye problems, and repository. The majority of waterborne diseases are life-threatening [3]. Primarily, children are victims of waterborne diseases due to poor hygienic water and weak immunity, especially since the condition is more aggravated in developing countries, including Pakistan. According to an estimate, 230,000 children die annually in Pakistan because of waterborne diseases [4]. According to the State Bank of Pakistan's report on health, included in the Sindh Vision 2030, water-related environmental health concerns impose the greatest health burden. In developing and transitioning nations like Pakistan, pathogens, including viruses, bacteria, and protozoa, transmit via the oral-fecal route and pose a grave risk of acute illness transmission via drinking water.

“Waterborne disease positive case detection” means detecting patients with waterborne diseases like malaria and typhoid. The health departments face the following challenges: (1) Tracing positive waterborne disease cases in different areas is challenging for health departments in underdeveloped countries. (2) One essential goal of the health departments is to find more affected areas in the country and find a type of gender with many positive cases. (3) present the waterborne disease information in a user-friendly interface so that the health departments can make rapid decisions.

Waterborne disease is one of the hot research topics nowadays. Most of the previous research has focused on predicting water quality indexes and forecasting water quality parameters*.* Moreover, various disease detection systems have been integrated into the health system to monitor positive cases [5, 6]. However, the prediction of efficient features for the positive cases of waterborne disease in Pakistan has yet to be addressed. However, most previous work uses statistical methods that are not easily generalized and interpretable. Therefore, the health departments need to use a waterborne disease intelligent detection system because detecting the correct positive cases of waterborne disease in different areas will decrease the number of deaths in the country. The challenge addressed in this study was determining the number of confirmed and fatal cases in the following week in various areas of Pakistan for the health authorities to make informed judgments. It identifies high-risk areas of Pakistan where additional cases are possible.

Machine Learning (ML) is an artificial intelligence (AI) concept that has been used to develop models or systems that reason with existing datasets to predict future events [7]. ML systems can read and change their structures based on observed data to achieve the desired objectives. ML is used to diagnose and predict diabetes, COVID-19, coronary artery disease, and waterborne diseases, just like other AI techniques like fuzzy logic, Data Mining (DM), and Deep Learning (DL). Numerous researchers have used ML approaches to forecast waterborne diseases for disease prevention. The authors of [5] created an ML model to simulate different sizes and lengths of waterborne disease outbreaks. More than 80% of the time, the model can predict how a disease will spread. Sandeep and Kuljit, in their study of a hybrid forecasting system for ANFIS, showed that a genetic algorithm-based model for cholera that spreads through water was used [8]. A system with ML Techniques for predicting the fecal contamination level of techniques been designed with the Artificial Neural Network at Recreational Beaches in Korea [9].

Support vector regression (SVR) was also applied to the Haeundae and Gwangallae Beaches in the Busan City dataset [10]. Studies based on statistical approaches, water quality, and photographs needed the interpretation and labeling of specialists.

This study has been designed as a web-based analytical dashboard for the health department. Different ML techniques have been designed as a framework for predicting a positive waterborne disease. The proposed method also predicts the attribute that plays an important role in predicting waterborne cases in Pakistan. The developed framework will help the health department in different areas of Pakistan.

### Motivations

This study's overarching objective is to develop a warning system or dashboard based on ML that enables the health department to obtain useful information from patient records, such as ranking the different types of waterborne diseases in various regions of Pakistan and then making predictions based on patient records. We explored the following research questions in this study.

1. Identifying the ML model with the greatest performance predicting positive cases based on patient history.

2. Discovering the input includes patient symptoms most relevant for patients with waterborne diseases.

3. Are all the waterways contaminated with waterborne sickness or clean?

4. Are there changes between the keywords used in research focusing on waterborne sample datasets released between 2015 and 2022?

5. Are there distinctions between the data analysis techniques used by research focusing on waterborne disease samples?

6. Are there discrepancies between the theories used in investigations of waterborne sickness samples?

### Research contributions

## *Contributions* The current study has contributed the following to our understanding of waterborne diseases:

- The current study employed actual patient data from Ayub Medical Hospital in Abbottabad, Pakistan, to forecast positive cases of waterborne disease using explainable ML techniques.
- Several ML classification methods were trained using a tenfold cross-validation method, and we discovered that RF is the most effective method for predicting positive cases of waterborne diseases.
- Using the RF technique, the contributing factors for predicting positive cases of waterborne disease were identified. To my knowledge, no research has been conducted to find the contributing factors.
- The current study's findings may be included in a web-based analytical dashboard system for predicting positive waterborne patients and identifying Pakistan's most affected regions.
- A fully automated novel model was developed for classifying waterborne diseases with the unique Ayyub hospital data.
- We have created a computationally efficient model using the Random Forest backbone. Due to the complexity of preserving computational power in ML architectures, the training process requires high computational power. In our case, we were able to train by decreasing the number of parameters after applying the Random Forest backbone in the encoder of the architecture.
- Our model is also the most time efficient during the training process. The model we used in our architecture assisted the model in converging faster than any other model we computed.
- Our architecture had the best output out of the other state-of-art architectures we compared in the same technical environment. This result was attained by applying dropout techniques.

## Related work

Significant research has been conducted using ML and statistical techniques to investigate waterborne diseases. Previous studies employed multiple ML approaches and input attributes to study the association between patient data and waterborne diseases.

For example, authors in [11] explained and compared strategies that may be used to estimate this relationship, drawing upon authoritative literature in the field. Mechanistic mathematical models benefit from being grounded in epidemiological theory, but they may need to incorporate crucial non-epidemiological factors that have an ambiguous relationship with human AMR. The authors recommend using panel regression models that may flexibly include these characteristics, capturing both form and scale variation. The authors propose suggestions for future panel regression research to inform cost-effectiveness assessments of AMR containment measures throughout the One Health spectrum, which will be key in an era of rising AMR.

Wan et al. [12] asserted that the pollution of drinking water supplies during floods is the primary source of waterborne diseases. Introducing bacteria, parasites, and viruses into the clean water system due to flooding results in the spread of waterborne diseases.

It has been found around the globe that the peak of waterborne epidemics occurred between 1980 and 2006, coinciding with the increase in the frequency of flood occurrences.

Eustace et al. [13] present a review of progress and advances made in detecting anomalies (Point anomaly, contextual anomaly, and collective anomaly) in water quality data using both traditional ML(ML) and DL approaches. Traditional ML methods are not as good as DL methods regarding how well features are learned and how often false positives happen. Nevertheless, it is hard to compare studies fairly because they use different datasets, models, and parameters. Therefore, this study also proposes a hybrid DL-ELM (deep learning-extreme learning machine) framework as a possible solution that could be investigated further and used to detect anomalies in water quality data.

Amy et al. [14] demonstrated a novel application of the effectiveness of random forest classifiers as an ML technique for cholera risk applications based on an analysis of seven remotely sensed Essential Climate Variables (ECVs) and their respective lagged values. In particular, they showed that the variables contributing most firmly to the Random Forest (RF) model cholera prediction included the one-month and two-month lagged values of chlorophyll-a concentration, sea surface salinity, land surface temperature, and sea level anomalies in the order of their contribution strength to the model performance results, respectively. Furthermore, it is noteworthy that while previous studies have utilized ECVs, this study demonstrates the first usage of remotely sensed sea surface salinity data in machine-learning analyses of a combination of ECVs to detect the risk of cholera outbreaks.

Authors in [15] analyze and forecast the values of water quality parameters to determine the concentration of Chlorophyll, Dissolved Oxygen, Turbidity, and Specific Conductance and analyze the results. The measurements of water quality parameters were scaled between 0 and 1 for better data handling. On the other hand, the artificial neural network (ANN) with nonlinear autoregressive (NAR) time series has been used with Scaled Conjugate Gradient (SCG) as a training algorithm. Four ANN models have been developed and analyzed, depicting the four selected water quality parameters. The performance measures used to depict the result are Regression, Mean Squared Error (MSE), and Root Mean Squared Error (RMSE). The proposed ANN-NAR model proves reliable, with the prediction accuracy indicating much-improved (0.99).

Zahra et al. [16] demonstrate the relationship between cholera incidence in Chabahar and the study variables, which were statistically evaluated. A designed artificial neural network model could predict cholera events up to 80% of the time. The results showed that neural networks could be useful tools for predicting infectious diseases, acting quickly to prevent them, reducing deaths, and stopping bad things from happening in society. Nevertheless, the prediction sensitivity needs to be higher because their dataset is smaller than they would like. Comprehensive data is necessary to achieve a more reliable prediction, and influential hygienic, social, and demographic parameters are recommended.

Ahmed et al. [17] built an ML classifier for predicting water quality in Indian rivers. After comparing different ML models (SVM, DT, KNN, NB method, and multi-layer perceptron), they observe that Multi-Layer Perceptron (MLP) gives the best results with 96.10% accuracy. They also observed that biochemical oxygen demand (BOD) is highly correlated with temperature because of oxygen depletion. On the other hand, dissolved oxygen (DO) is negatively correlated with temperature; therefore, DO has little variation during the summer.

On the other hand, Juan et al. [18] compared Support Vector Machines (SVM), Artificial Neural networks (ANN), K-nearest neighbors (KNN), and a Decision Tree (DT) Regressor in addition to two linear approaches. With this, they obtain an operational methodology contributing to the Argentinean Dengue Risk System. Furthermore, they explore the ability to model and predict oviposition without the shelf ML algorithms, i.e., with minimum parameter tuning, as provided by FLOSS—Free/Libre Open-Source Software. This promotes the assimilation of these techniques for the whole community that deals with similar problems.

Mu et al. [19] employed FT-NIR spectroscopy to discriminate against waterborne pathogenic bacterial strains. To achieve good classifications, they compared different spectral transforms and preprocessing methods. As a result, the best PLS-DA (partial least squares discriminant analysis) classification model was achieved based on the original transmittance spectra. The overall correct classification rate for this model was 70.4% for prediction. Moreover, nonlinear methods such as SVM and radial basis function neural networks (RBF) were also utilized to optimize classification performance, where excellent overall correct classification rates (OCCRs) (> 96%) were attained. In developing simplified classification models, successive projection algorithms (SPA) and competitive adaptive reweighted sampling (CARS) were applied to identify important wavelengths for bacterial classification. The results showed that CARS and SVM produced the best OCCR of 100% for prediction.

Hatice et al. [20] developed a hand-held and cost-effective platform for automated detection and counting of Giardia cysts in large water samples using machine learning. This platform includes a mobile phone-based fluorescence microscope to capture an image fluorescently. This fluorescence image of the sample filter membrane, captured using a custom-developed application, is processed at their servers for automated detection and enumeration of Giardia cysts using a training image dataset. Comparing many supervised machines learning classification models, they proved that this platform could detect and count Giardia cysts in water samples with 95% classification accuracy.

Yongeun et al. in [10] predict the current level of fecal contamination (enterococcus [ENT] and Escherichia coli) using readily available environmental variables. Artificial neural network (ANN) and support vector regression (SVR) models were constructed using data from the Haeundae and Gwangalli Beaches in Busan City. The performance of the ANN model for predicting ENT and E. coli at Gwangalli Beach was significantly higher. The ANN demonstrated better performance than the SVR. This study suggests an effective prediction method to determine whether a beach is safe for recreational use.

Phong et al. [21] assess the groundwater potential of the DakNong province, Vietnam, using an advanced ensemble ML model (RABANN) that integrates ANN with the Real AdaBoost (RAB) ensemble technique. Twelve conditioning factor datasets were used to create the training and testing datasets to develop and validate the ensemble RABANNE model. They demonstrated that the RAB ensemble technique was successful in improving the performance of the single ANN model. As a result, the study would help improve the area's groundwater, which would help eliminate health problems caused by diseases that spread through water.

Pratima et al. [22] **t**ransform existing health services into a modern, high-performing, quality health system. Also, the ministry wants to use information technology to its fullest extent to help people live. The dataset was then used to train an artificial neural network ML algorithm, which could then predict the propagation of the diseases using weather forecasts. The dataset was then used to train an ML algorithm called an artificial neural network. The algorithm could then use weather forecasts to predict how diseases spread. The accuracy of 90% indicates that such a system is viable and can be a good candidate for an event-based detection system and help the ministry achieve its objectives.

Umair et al. [23] explored an alternative method of ML to predict water quality using minimal and easily available water quality parameters. The data used to conduct the study was acquired from PCRWR and contained 663 samples from 12 different sources in Rawal Lake, Pakistan. A set of representative supervised ML algorithms was employed to estimate WQI. This showed that polynomial regression, with a degree of 2, and gradient boosting, with a learning rate of 0.1, outperformed other regression algorithms by predicting WQI most efficiently, while MLP, with a configuration of (3, 7), outperformed other classification algorithms by classifying WQC most efficiently.

Chen et al. [24] review the current status of watershed science for both water quantity and quality and identify critical gaps in our current knowledge and modeling capability to address the emerging needs in predicting watershed hydrologic and biogeochemical responses to natural and anthropogenic perturbations. They emphasize the need to comprehend how environmental perturbations, such as floods and droughts, and anthropogenic changes, such as deforestation and urbanization, propagate through watershed systems and evaluate their short- and long-term effects on watershed biogeochemistry, water quality, and their recovery pathways.

Archana et al. [25] built a system that continuously monitors water quality. It can be helpful to monitor the quality of water in any uncertain condition using ML methods, techniques, and input features used in related work to investigate waterborne diseases.

The major conclusions derived from the literature study are outlined below:

- The study’s results conclude that variation can be predicted at an acceptable accuracy rate using unsupervised learning and data with work.
- Results show that Turbidity has high variability compared to the other two parameters but is now low. It is affected most during the monsoon season.
- pH has little variation in data; hence, it is stable compared to Turbidity and DO.
- DO has little variation during the summer as the temperature affects the water quality during the summer.

As seen previously, many studies have been done on the same topic with supervised and unsupervised methods, though most have an acceptable accuracy rate. Additionally, we have enhanced the accuracy and ROC curve. However, most previous work uses statistical methods that are not easily generalized and interpretable, and previous studies use images or input features unrelated to real-world challenges. Therefore, we used real environment data to investigate the current study.

## Methodology

### Data collection

In this research, malaria and typhoid patients’ clinical data were formally collected from Ayub Medical Hospital. The clinically collected data were from 4 years (2017–2020). This research study was conducted on waterborne typhoid and malaria-infected patients at different urban locations in Khyber Pakhtunkhwa (KPK). The typhoid data comprised 68,624 unique patients, of whom 32,315 were males, 36,283 were females, and 26 were neuters. These patients had visited the hospital for diagnosis and to obtain medical treatment.

Similarly, malaria data comprised 22,916 unique patients, out of whom 12,120 were males, 10,793 were females, and 3 were neuters. The variables used in this investigation were painstakingly retrieved from hospital-patient databases. A detailed description of each variable of data is mentioned in Table 1.

**Table 1:** Input features and details of the current study

Variable name	Data description
Age	Age of the patient when came for diagnosis
Gender	Male/Female/Neuter indicator of patient
District	District from where patients belong to
Tehsil	Sub-division from where patients belong to
MRNO	A Medical Record Number (MRN) is a healthcare organization-specific identifier given to each patient upon the arrival of the first visit
REPORT_VERIFIED	Date of the test report uploaded to the system
CPT_ID	Current Procedural Terminology (CPT) ID that were used to identify medical services and the procedures
RESULT_VALUE	Result Value Y shows the process of the test report is complete, and the result value is available; and N shows the incomplete test reports
RESULT_TEXT	Value of test result i-e Negative or Positive

As seen in Table 1, age, gender, district, tehsil, MRNO, Report Verified, CPT ID, Result Value, and Result text are the study's input variables. The patient's age depends on the aquatic illness since waterborne diseases mostly affect younger patients. District and Tehsil indicate the country's region, and waterborne diseases are also affected by region.

### Proposed architecture

**Fig. 1:**
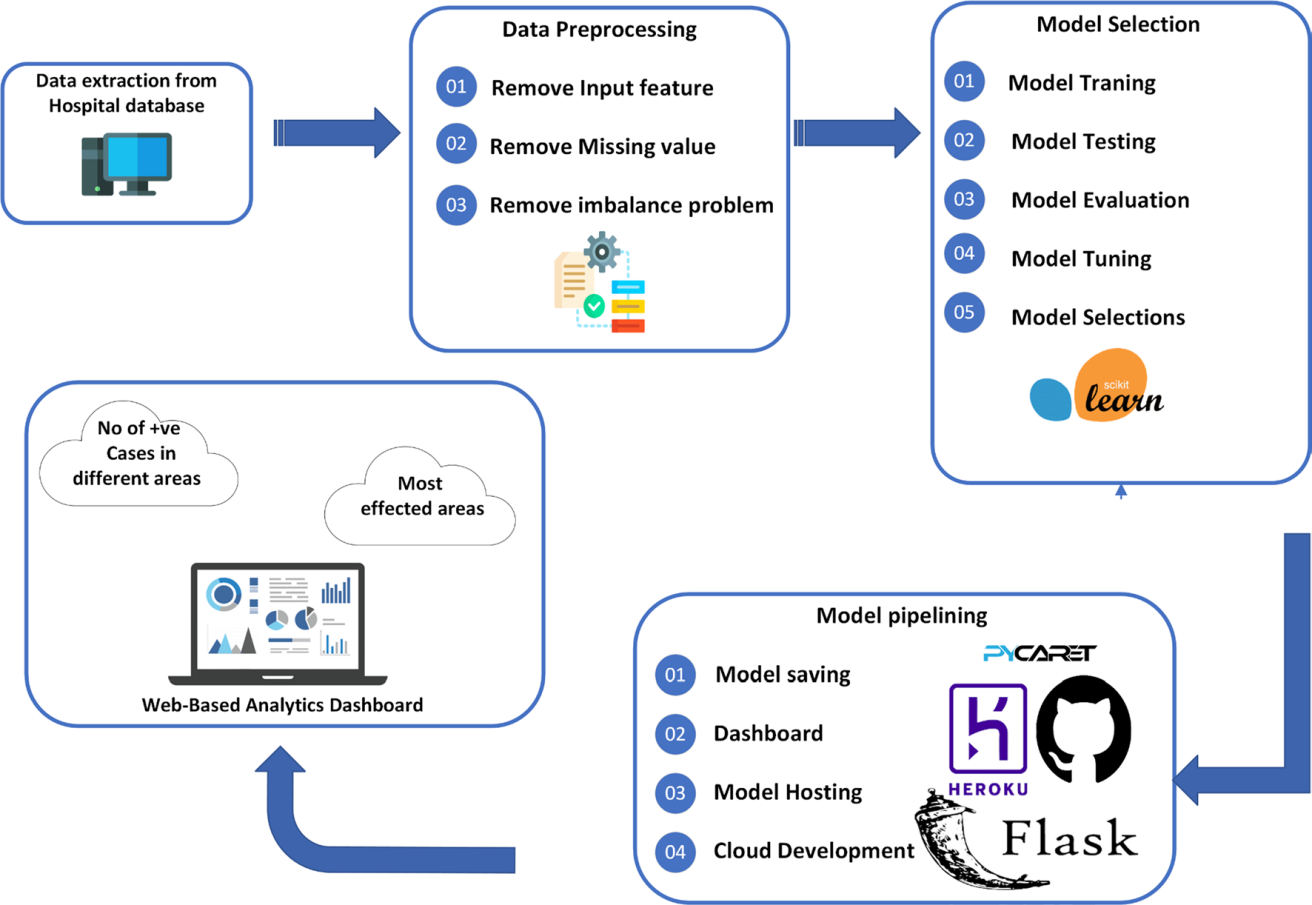
Proposed framework for waterborne disease

Waterborne disease is investigated in this study using machine techniques. Figure 1, mentioned below, shows the proposed framework for waterborne disease. The proposed framework has the following phases.

#### Data Preprocessing

We collect raw data about malaria and typhoid. We perform the following preprocessing procedures using the Python library to convert the data into a format suitable for the present research model. *Data cleaning* The original data contains missing values; consequently, the median imputation approach was used to fill in the missing values since it is simple, efficient, and changes the statistical structure of the input features.

*Imbalance problems* As in our dataset, the number of observations differs for the class in the classification dataset, so our dataset suffers from an imbalance problem. The imbalance problem can lead to inaccurate results. Also, in the absence of a good-quality dataset, we will only be able to produce good results if we perform experiments with the best algorithms. The imbalance problem in our dataset meant that a dataset is biased towards a class in the dataset, and if it's biased towards one class, then an algorithm trained on the same data will also be biased toward the same class. So, we need to solve the data imbalance problem, and we have used resembling techniques to mitigate the problem [26].

*Data Transformation* Data transformation is a crucial step in data preprocessing. After cleansing the data, categorical data were transformed into a numerical representation using the hot encoding approach since most ML algorithms do not handle categorical data.

*Data normalization* Additionally, final data were normalized using the z-score technique because the ML model does not process the un-normalized data.

#### Model selection

After data preprocessing, the dataset was ready for training in the current study's ML model. First, we trained and tested five ML models on waterborne disease datasets using the Sklearn Python library. Secondly, higher-performance models are saved for integration in the web app.

#### Analytical dashboard

In a further phase**,** an analytical dashboard (warning system) for the waterborne disease has been developed using the best ML model using the Python framework (Flask and Pycaret), shown in Fig. 1. The current study dashboard will detect positive cases and visualize the information on waterborne disease patients in different areas. The proposed framework will also be used to find the most affected areas. Table 1 shows the attributes used in the Malaria and Typhoid dataset.

#### Mathematical formulation

The mathematical formulation of our proposed articulation is as follows. The data set collected malaria S1 and typhoid S2 patients. From 2017 to 2020, 22,916 patient samples were collected for malaria (n1), whereas 68,624 patient samples were collected for typhoid (n2) from 2017 to 2020. Seven input features (P) were used to predict positive cases of waterborne disease. A detailed list of attributes used in the malaria and typhoid dataset is mentioned in Table 1. P + 1 shows the response variable, and Y indicates resutl_text. The response variable Y depends on predictor variables P. Then we combine the attribute of P-dimension in Vector, which shows below:

1 $$X1 = \left[ {X1,X2 \ldots Xp} \right]$$

2 $${\text{X}}2 = \left[ {{\text{X}}1,{\text{X}}2 \ldots {\text{Xp}}} \right]$$

Further, $Y \sim \left[ {y1,y2} \right]y1 = 1$, which indicates a positive class of malaria and typhoid disease, and **y = 0,** which indicates a negative class of malaria and typhoid disease. Then we study classification problems by applying different ML classification algorithms to the current study dataset. During the training process, we take a vector of input feature with corresponding Y (training dataset) and then find the function called prediction function or model, which associates class and label with every vector as below.

3 $$y = f\left( X \right) + w$$

For unseen samples where X = z, the proposed framework used trained model **f** to predict the class and label of unseen samples. This study investigated the performance of Random Forest [27], LR [28], DT [29], Support Vector Machine [30], and KNN [31] on the current study dataset.

## Experiment and discussion

In this portion of the paper, we predicted the number of positive cases of waterborne disease patients from patients' records using input features related to patients. To explore the research questions of this study, we conducted many experiments. We used different ML algorithms and Python modules to develop the learning models described below.

### Data visualization

**Fig. 2:**
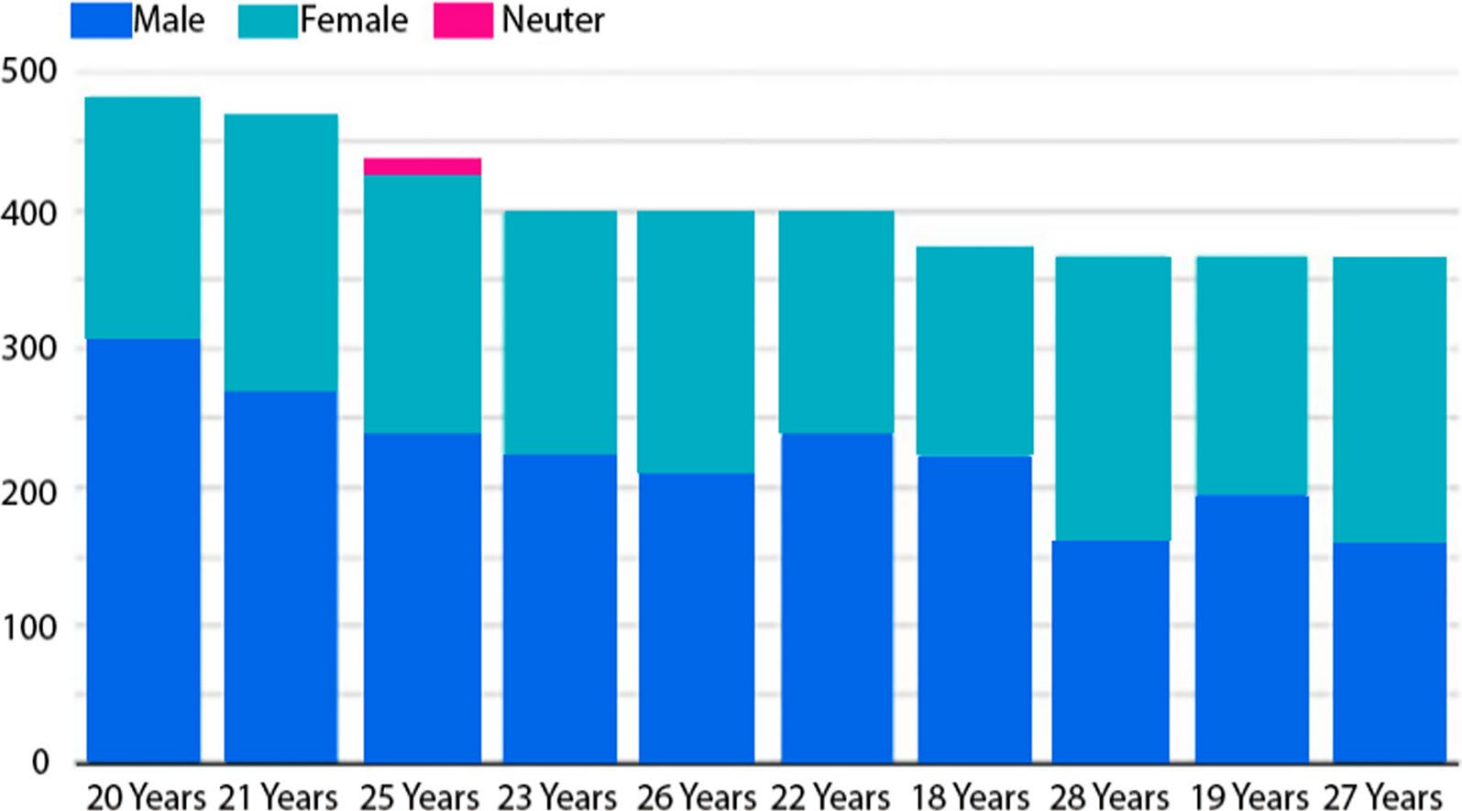
Data Visualization between positive cases of malaria and gender

In data visualization, we plot relationships between different input features to explore further and target variables. For example, Fig. 2 shows data visualization between positive malaria cases and gender.

**Fig. 3:**
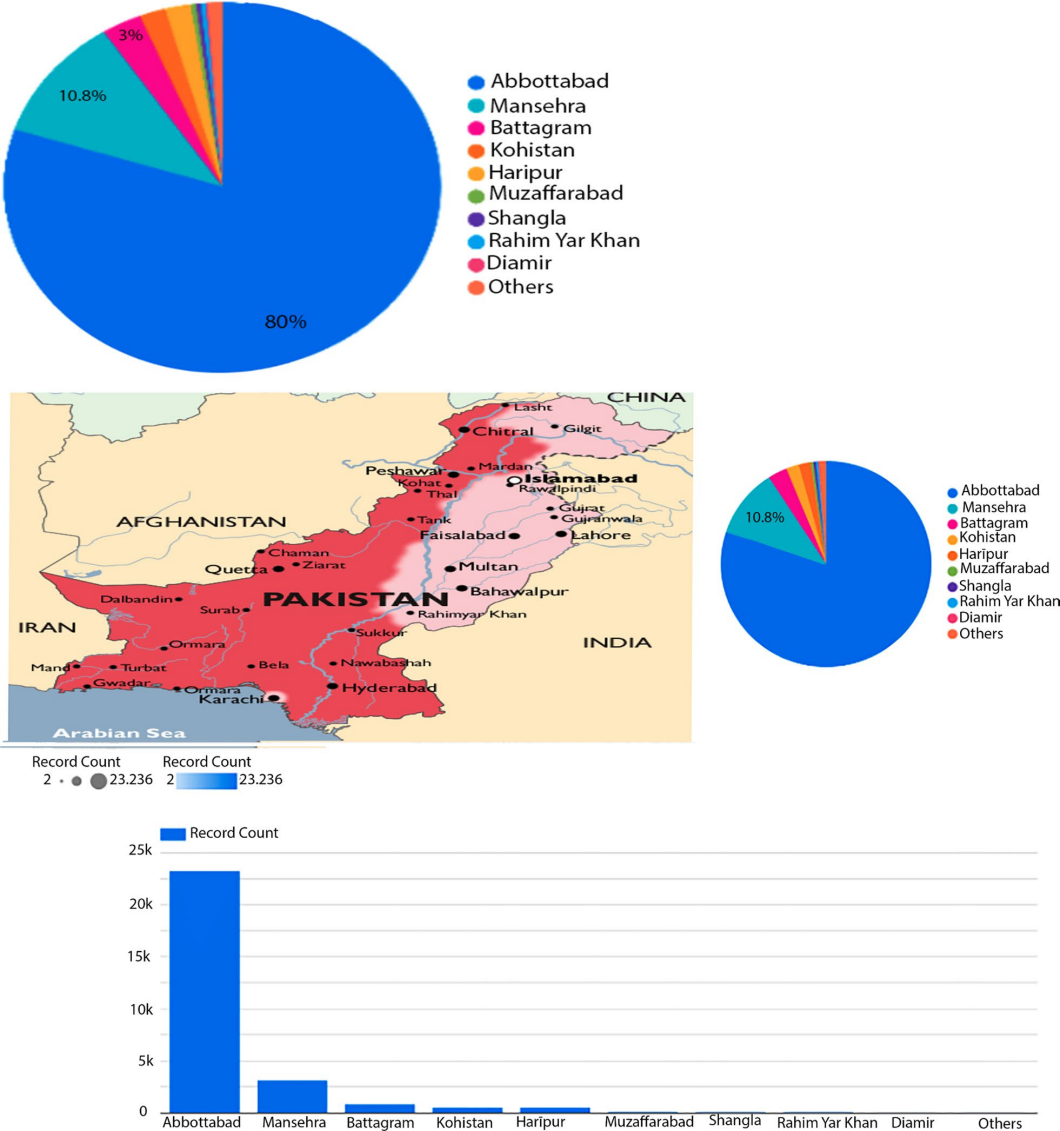
Data visualization between malaria-positive cases and area of Pakistan

Figure 2 shows that 20-year males and females are more affected due to positive malaria cases compared to other age of patients. Figure 3 displays data visualization between malaria-positive cases and the area of Pakistan.

As seen in Fig. 3, Abbottabad and Mansehra are the most affected areas compared to other areas of Pakistan.

### Experiments

Waterborne disease detection is an important issue for the country and the health department**;** therefore**,** this section investigates the number of positive cases of patients in the hospital database using ML models. To investigate waterborne disease, we explored the following research questions:

*Question 1* Identify an effective ML model with the highest performance predicting positive cases using patient history.

To explore this question, we conduct the first experiments using the Python Sklearn library to detect positive cases of waterborne disease using patient records. To explore the research questions of this study, we conduct several experiments. First, we used the Common ML algorithms (DT), Random Forest (RF), Support vector machine (SVM), Logistic regression (LR), and K-Nearest Neighbor (KNN) to predict the waterborne disease-positive cases. These algorithms are easily explainable, interpretable, implemented, and used in many fields with good performance f, such as education and medicine. Furthermore, these algorithms require fewer data and are less computationally expensive to train than the artificial neural network and another DL algorithm. To increase the model's generalization ability on the test data, we train and test the model on 10- fold cross-validation**.** The DT, RF, LR, KNN and SVM algorithms predict positive patient cases using the patient records.

As is known, SVM was originally mentioned in 1992 when Boser, Guyon, and Vapnik described it in COLT-92. SVMs are a group of supervised learning techniques used to classify and predict. They are related to generalized linear classifiers. SVM is an alternative to a tool for predicting classification and regression. It uses ML theory to improve the accuracy of predictions without overfitting the data. SVMs are systems that employ the hypothesis space of linear functions in a high-dimensional feature space and are taught using an optimization-based learning algorithm that applies a statistical learning bias. SVM was originally well-liked by the NIPS community and are now an integral aspect of ML research worldwide.

On the other hand, DT in classifier-generating systems is one of the most used DM approaches. In data mining, classification algorithms can manage huge quantities of data. It may establish assumptions about categorical class names, categorize knowledge based on training sets and class labels, and classify freshly obtained data. In machine learning, classification methods include several algorithms; however, this study focuses on the DT approach—RF created by Breiman to tackle classification and regression issues using ensemble learning. Ensemble learning is a technique for ML that improves accuracy by combining many models to address the same issue. Specifically, ensemble classification uses many classifiers to produce more accurate results than a single classifier. RF belongs to the ensemble algorithms category, and it is a tree-based, effective, supervised algorithm and an easily understandable and interpretable algorithm. Furthermore, it predicts using a collective method by creating multiple DTs. It is also called the ensemble method because it creates multiple models and combines them into a single model.

While LR is a popular statistical model that allows for multivariate analysis and modeling of a dependent variable with two possible values, linear regression is a similar model for a dependent variable with a single value. The multivariate analysis estimates coefficients for each predictor in the final model and adjusts them with the other predictors in the model. Also, in pattern recognition, KNN is a well-known non-parametric classifier that is commonly used. The k-nearest neighbors' method, often known as KNN or k-NN, is a non-parametric, supervised learning classifier that employs proximity to create predictions or classifications about the grouping of a single data point.

Among all these mentioned techniques, we created the ML models with Python’s Sklearn library and tested their accuracy with tenfold cross-validation. This study uses accuracy and the ROC curve as evaluation matrices. These metrics are defined below:

4 $${\text{Accuracy }} = \frac{{{\text{TP}} + {\text{TN}}}}{{{\text{TP}} + {\text{TN}} + {\text{FP}} + {\text{FN}}}}$$

Equation 1 shows where TP = true positive; FP = false positive; TN = true negative; and FN = false negative.

In addition, the roc curve plots the true positive rate again against the false positive rate of the model.

The DT is a tree-based, widely used technique for classification problems in different fields, for example, medical [38] and banking [39]. It can learn from a small dataset [32]. We trained and tested the DT model on the current study dataset. Table 2 shows the confusion matrix of DT on malaria, and typhoid datasets, respectively.

**Table 2:** Confusion matrix of the decision tree classifier on the Malaria dataset

	Positive (Actual)	Negative (Predicted)
Positive (Actual)	TP = 5189 (0.64)	FP = 3497 (0.46)
Negative (Predicted)	FN = 2864 (0.36)	TN = 4058 (0.54)

Table 2 depicts the confusion matrix of the DT classifier for Malaria, where TN stands for true negative and refers to the number of predictions in which the classifier correctly predicted the negative class (case) as negative. False negative (FN) is the number of times a classifier wrongly predicts that a positive class is negative. FP stands for "false positive," which is the number of times the classifier gets it wrong and thinks the negative class is positive. At the same time, TP stands for "true positive" and refers to the number of predictions in which the classification algorithm properly predicts the positive class as positive, just as DT is a general approach for classifying difficulties. On the malaria data set, the result shows that the DT was about 59% accurate. Table 3 shows the confusion matrix of DT on malaria, and typhoid datasets, respectively.

**Table 3:** Confusion matrix of the random forest classifier on the Malaria dataset

	Positive (Actual)	Negative (Predicted)
Positive (Actual)	TP = 2593 (0.58)	FP = 430 (0.07)
Negative (Predicted)	FN = 1903 (0.93)	TN = 5441 (0.93)

The Confusion matrix of the random forest classifier on the typhoid dataset is shown in Table 4.

**Table 4:** Confusion matrix of the random forest (RF) classifier on the typhoid dataset

	Positive (Actual)	Negative (Predicted)
Negative (predicted) FP = 430 (0.07)	Negative (predicted) FP = 430 (0.07)	Negative (predicted) FP = 430 (0.07)
Negative (predicted) FP = 430 (0.07)	Negative (predicted) FP = 430 (0.07)	Negative (predicted) FP = 430 (0.07)

From Table 4, the true positive rate (malaria dataset = 0.58, typhoid dataset = 0.58) seems quite high when the confusion matrix is checked. On the other hand, the mispredicted values (FP) (malaria dataset = 0.07, typhoid dataset = 0.07) seem fairly low. This proves that the model works quite well. The result indicates that the RF achieved an accuracy of 0.60 on the malaria dataset. in comparison to 0.775 for the Typhoid dataset. SVM is also used to solve the supervisor problem and transfers input features in high dimensional space. The SVM predicts malaria and positive typhoid cases with an accuracy of 0.58 and 0.60, respectively. Table 5 shows that the TP rate of SVM on malaria and typhoid datasets are 0.13 and 0.67, respectively. The FP rate of SVM on malaria and typhoid datasets are, respectively, 0.1 and 0.5. Table 5 displays the Confusion matrix of the Support Vector Machine.

**Table 5:** Confusion matrix of the Support Vector Machine Classifier on the Malaria dataset

	Positive (Actual)	Negative (Predicted)
Positive (Actual)	TP = 590 (0.13)	FP = 45 (0.01)
Negative (Predicted)	FN = 3906 (0.87)	TN = 5826 (0.99)

The Confusion matrix of the Support Vector Machine classifier typhoid dataset is shown in Table 6. In addition, the TP rate of SVM on malaria and typhoid datasets is 0.13 and 0.67, respectively.

**Table 6:** Confusion matrix of the Support Vector Machine classifier typhoid dataset

	Positive (Actual)	Negative (Predicted)
Positive (Actual)	TP = 5362 (0.67)	FP = 3771 (0.50)
Negative (Predicted)	FN = 2691 (0.33)	TN = 3784 (0.50)

As seen, Table 6 proves that the correctly identified values by SVM are quite high, especially the true positive rate. The misclassified values are low compared to the typhoid dataset. Table 7 shows the confusion matrix of the Support Vector Machine on the typhoid dataset**.** Also, a commonly used classification algorithm that finds the relationship between dependent and independent variables. The LR predicts malaria-positive cases with an accuracy of 0.58 and typhoid-positive cases with an accuracy of 0.615.

**Table 7:** Confusion matrix of the logistic Regression classifier on the Malaria dataset

	Positive (Actual)	Negative (Predicted)
Positive (Actual)	TP = 5369 (0.67)	FP = 3771 (0.50)
Negative (Predicted)	FN = 2684 (0.33)	TN = 3784 (0.50)

As seen below the Table 8 show the confusion matrix of LR on malaria and typhoid datasets.

**Table 8:** Confusion matrix of the logistic Regression classifier typhoid dataset

	Positive (Actual)	Negative (Predicted)
Positive (Actual)	TP = 1398 (0.31)	FP = 891 (0.15)
Negative (Predicted)	FN = 3098 (0.69)	TN = 4980 (0.85)

Table 8 show that the LR, TP rate on malaria and typhoid dataset are 0.67 and 0.31, respectively Thus, on the other hand, the FP rate on typhoid and malaria dataset are 0.50 and 0.15, respectively.

From 9, LR on the malaria dataset does not work well enough to identify. The accuracy rate is 58%. The KNN algorithm is an easy-to-understand ML algorithm used for supervised problems. It received an accuracy of 0.59% on the malaria dataset during the testing phase and received 0.61% accuracy on the Typhoid dataset. Tables 9 and 10 show the confusion matrix results of KNN on malaria and Typhoid dataset. The True Positive rate (0.70) and False Positive rate (0.52) of KNN on the malaria dataset are shown in Table 10.

**Table 9:** Confusion Matrix KNN Algorithm on Malaria dataset

	Positive (Actual)	Negative (Predicted)
Positive (Actual)	TP = 5648 (0.70)	FP = 3919 (0.52)
Negative (Predicted)	FN = 2405 (0.30)	TN = 3636 (0.48)

**Table 10:** Confusion Matrix KNN Algorithm on typhoid dataset

	Positive (Actual)	Negative (Predicted)
Positive (Actual)	TP = 642 (0.14)	FP = 132 (0.02)
Negative (Predicted)	FN = 3854 (0.86)	TN = 5739 (0.98)

As seen, Table 10 displays Confusion Matrix KNN Algorithm on typhoid dataset. The results are promising. In Table 10 True positive rate (0.14) and false positive rate (0.02) of KNN on Typhoid dataset.

**Fig. 4:**
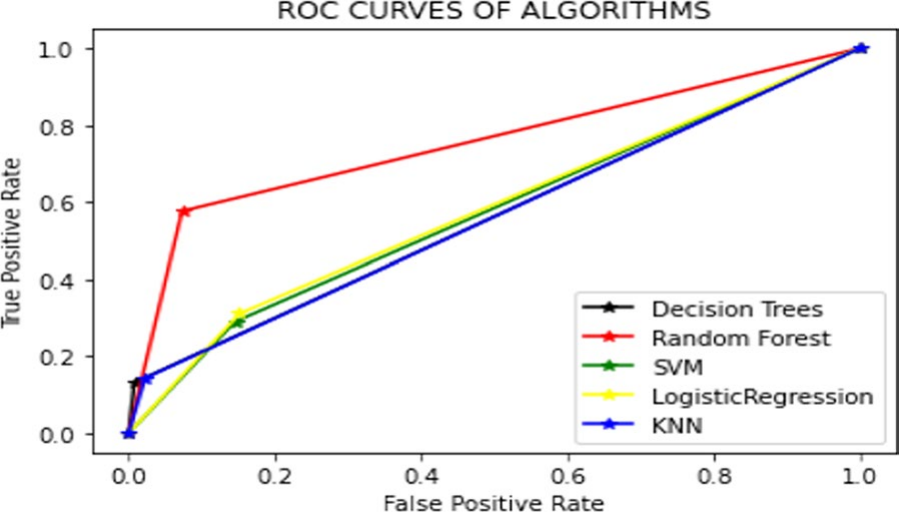
Comparison of ROC curve of all algorithms on the malaria dataset

As seen in Table 10, KNN Algorithms on the typhoid dataset are working with failure on the malaria dataset. Finally, Fig. 4 shows the ROC comparison of all algorithms on the malaria dataset. Furthermore, Fig. 4 compares the ROC curve of all algorithms on the malaria dataset.

**Fig. 5:**
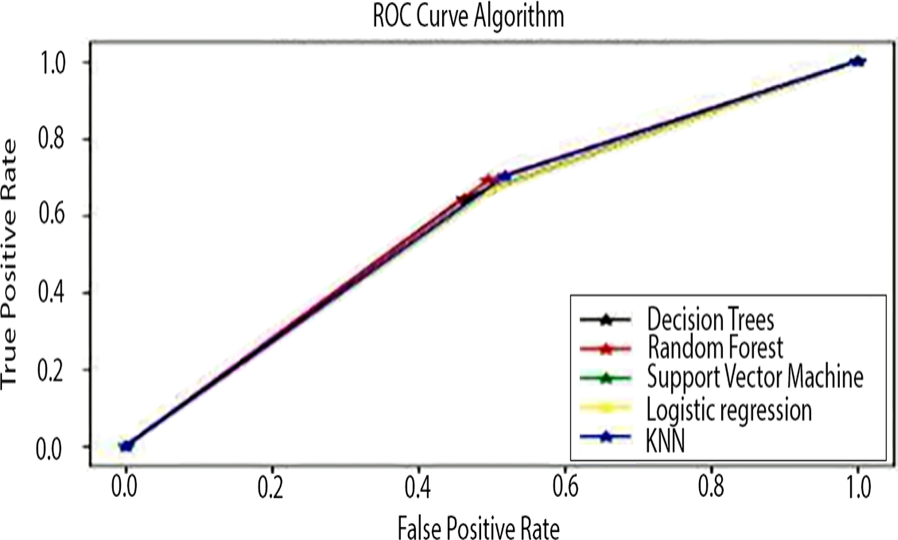
Comparison of ROC curve of all algorithms on typhoid dataset

Figures 4 and 5 are about the ROC curve, which displays DT, RF, SVM, LR, and KNN performance on the malaria dataset; as seen, RF shows the highest performance on the malaria and typhoid datasets.

As seen from Fig. 5 shows that although most models are close to each other, RF proves the highest performance. Table 11 illustrates Compare the accuracy of all algorithms on the malaria and typhoid datasets.

**Table 11:** Compare the accuracy of all algorithms on malaria and typhoid datasets

Model		Accuracy
*Comparison of all algorithm results malaria dataset*		
Decision tree algorithm Random forest algorithm Support vector machine algorithm Logistics Regression algorithm KNN algorithm		0.5925 0.6003 0.586 0.5864 0.5948
*Comparison of all algorithms results typhoid dataset*		
Decision tree algorithm Random forest algorithm Support vector machine algorithm Logistics Regression algorithm KNN algorithm	0.6189 0.775 0.6095 0.6152 0.6155	

Using correlated characteristics, Table 12 compares the accuracy of all algorithms on malaria and typhoid datasets.

**Table 12:** Compare the accuracy of all algorithms on malaria and typhoid datasets using correlated features

Model		Accuracy
*Comparison of all algorithm results malaria dataset*		
Decision tree algorithm Random forest algorithm Support vector machine algorithm Logistics Regression algorithm KNN algorithm		0.6421 0.**7453** 0.5779 0.5779 0.5789
*Comparison of all algorithms results typhoid dataset*		
Decision tree algorithm **Random forest algorithm** Support vector machine algorithm Logistics Regression algorithm KNN algorithm	0.5982 0.601 0.5957 0.5956 0.597	

**Fig. 6:**
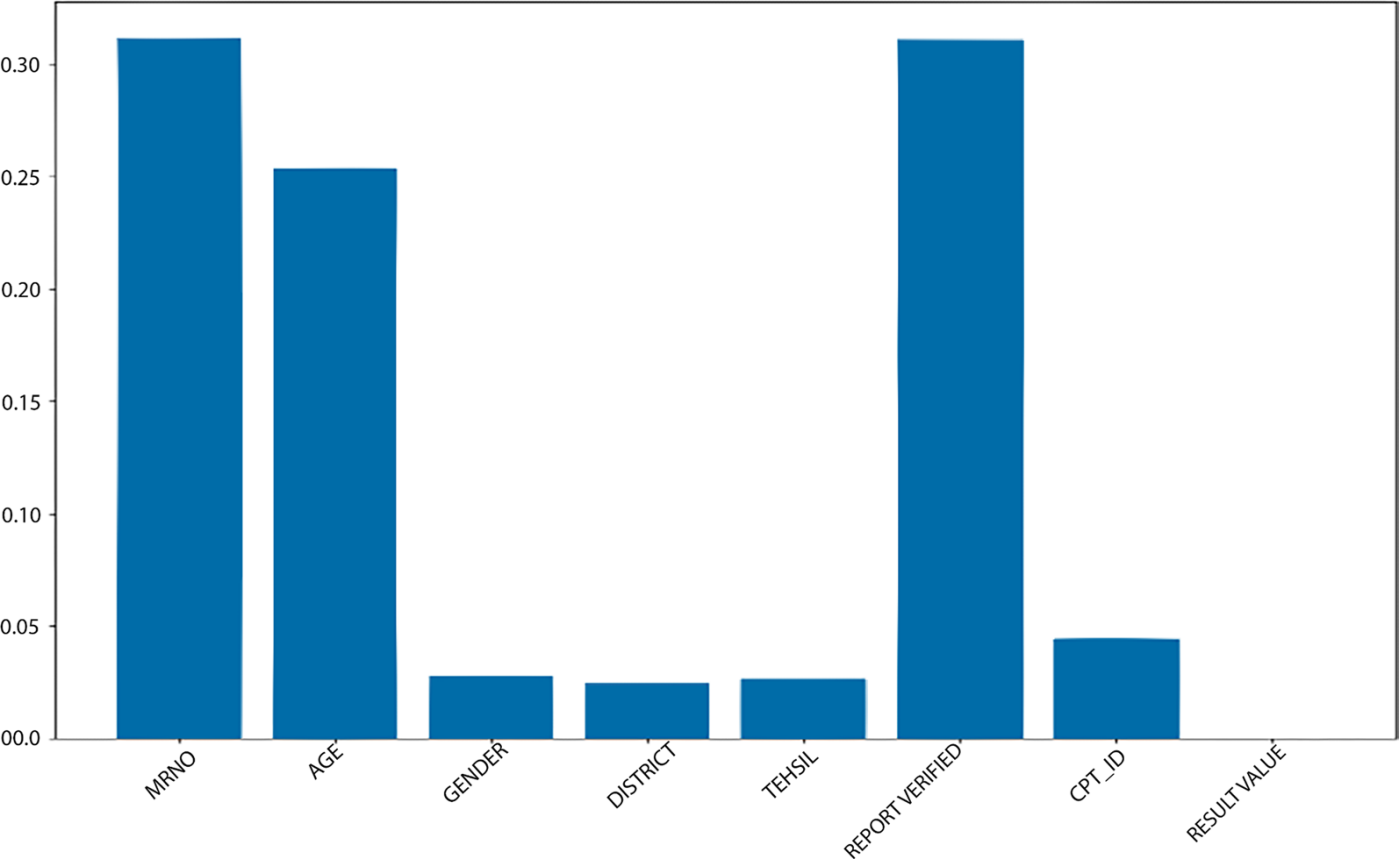
The histogram shows the importance of each feature of the Malaria Dataset

Table 11 shows DT, RF, SVM, LR, and KNN performance on the malaria and typhoid datasets using all input features. Moreover, DT, SVM, LR, and KNN perform well in other domains. But the result (Table 12) shows that RF received high accuracy and high ROC (as shown in Figs. 4 and 5) value on the malaria and typhoid dataset compared to the other models DT, SVM, LR, and KNN. Table 12 displays the performance of the current study's ML model employing significant features; the results indicate that the performance of RF on the malaria dataset is superior to that of the typhoid dataset. Figure 6 displays the histogram shows the importance of each feature of the Malaria Dataset.

**Fig. 7:**
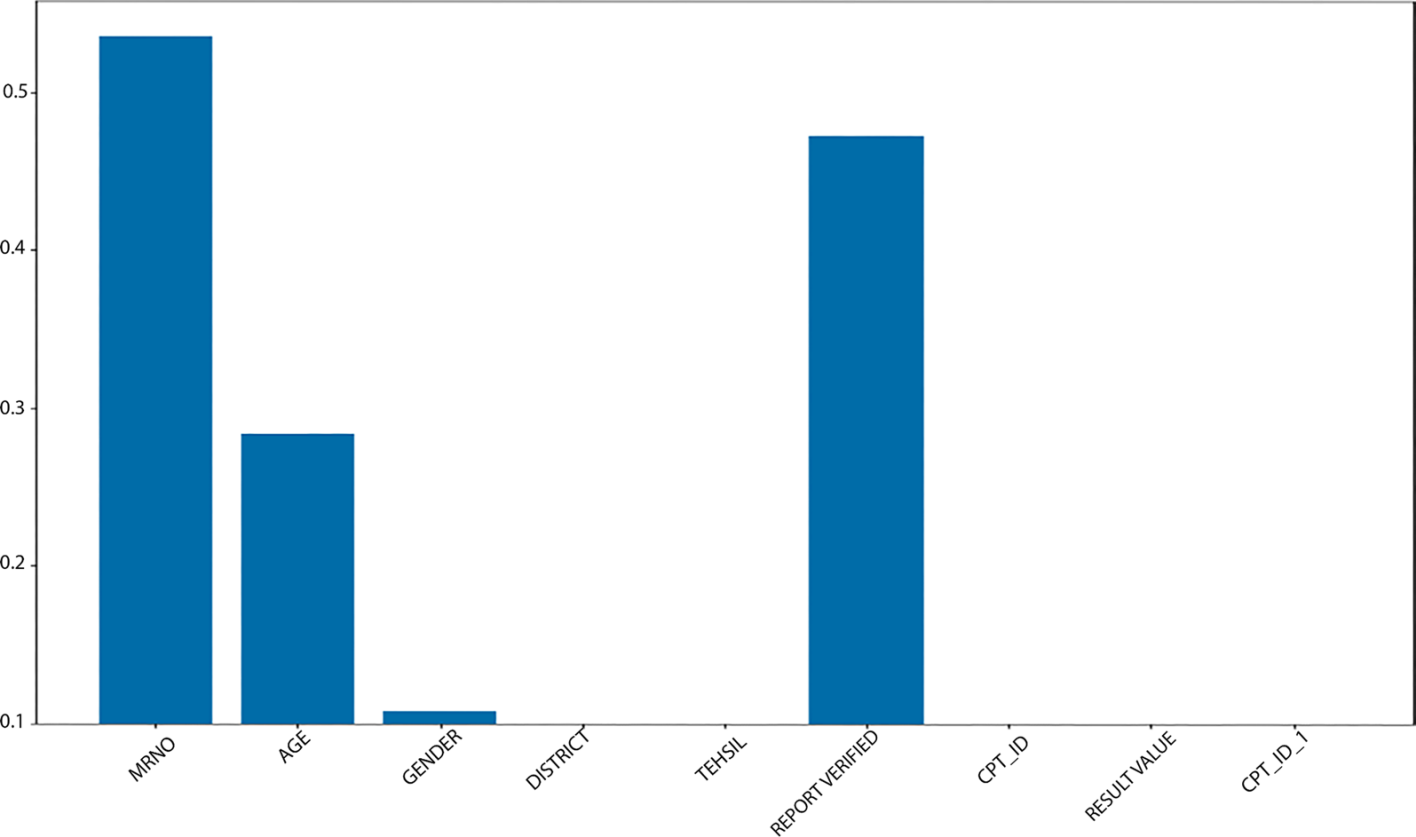
The histogram shows the importance of each feature of the Typhoid Dataset

As seen from Fig. 6 Age parameter is about 0.25 while Gender is under 0.05. Also, Fig. 7 shows the histogram shows the importance of each feature of the Typhoid Dataset.

*Question 2* Discovering the features of patients that are most significant for waterborne disease.

To explore Question 2, we performed a second experiment. In this experiment, we used the random forest feature selection technique. RF is a very efficient supervised algorithm, usually predicting required classes with high accuracy. Using RF, we can also find the features' importance during prediction. The RF feature selection technique was used in the second experiment to determine how waterborne disease symptoms are associated with waterborne disease-positive cases. We discover that patients have MRNO and report highly related, verified cases of water-borne positivity, as shown in Figs. 6–7. First, the age factor shows that most children and old people are more affected by waterborne diseases. Second, MRNO features show that a patient's history is a big part of determining if they will get a waterborne disease. Third, the report verified that a patient laboratory test is crucial for predicting waterborne disease.

### Discussion

This study evaluates how well a standard ML classification method works on patient data about waterborne infections. In earlier research, the illness known as water bronchitis was investigated. Two hundred and eleven different seasonal climate datasets were used in the study that Kim et al. [26] and Wang et al. [27] conducted on malaria. However, most research is conducted on visual or input elements that have little to do with the real world's challenges. ML algorithms are now the subject of a substantial amount of research currently being conducted to explore waterborne illnesses such as malaria and typhoid. Considering this, for the sake of research in this study, we used data on waterborne illness outbreaks from a hospital that was open to the public. In Pakistan, malaria and typhoid are two of the most significant waterborne infections; as a result, patient data for malaria and typhoid were utilized in this study. We used the medical data of 22,916 people with malaria to train and evaluate the ML models.

According to the obtained findings, RF performed much better than other ML models illustrated in the malaria dataset, as shown in Table 11,12. Because precision might occasionally result in an incorrect prediction of the outcome, we produced the ROC curves for all ML techniques. We plotted them in Fig. 4,5 using malaria and typhoid dataset. Figure 4,5 demonstrates that RF has a good ROC value and may reliably identify instances of positive malaria. The solution uses an under-sampling approach with a Random Forest (RF) algorithm. This approach does not rely on heuristics and is often considered the quickest, most straightforward option for dealing with enormous datasets. This strategy eliminates class instances from the majority class using a random selection process to achieve the desired distributional balance. However, since this is a random strategy, the relevant data might be eliminated from the majority class, which is important information for classifiers. However, this method works well with more extensive datasets [28, 29]. In addition, the under-sampling method is appropriate for eliminating overlapping data points in the class that constitutes the majority [33].

The instances closer to the decision boundary between two classes are considered more important to the classification than the examples that are farther away from the boundary. Most of these instances on edge are Support Vectors, which are important elements of SVM Classification. Unlike non-support vectors, SVMs can evaluate which hyperplane parameters are more important in preserving important information and avoiding accumulating irrelevant information. However, there is a possibility that SVs will favor members of a disadvantaged class [24, 37]

KNN is a method that is reliable, easy to comprehend, and accurate, and it requires the adjustment of only a few parameters. In addition, there is a low entry barrier when updating KNN to the new training instance. However, KNN requires a lot of processing power and only considers the local prior probability when making predictions [38–40].

**Fig. 8:**
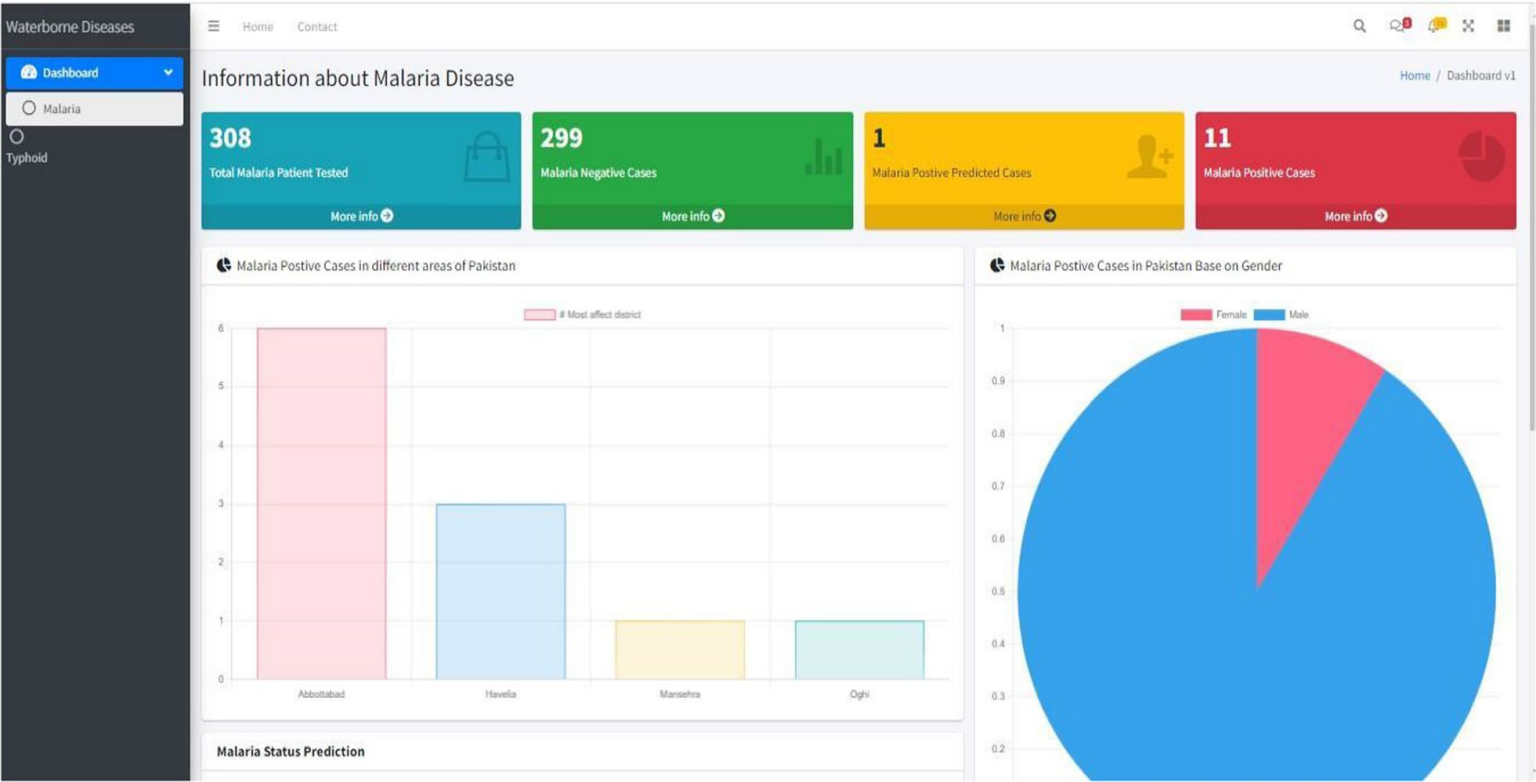
Waterborne disease dashboard snapshot 1

**Fig. 9:**
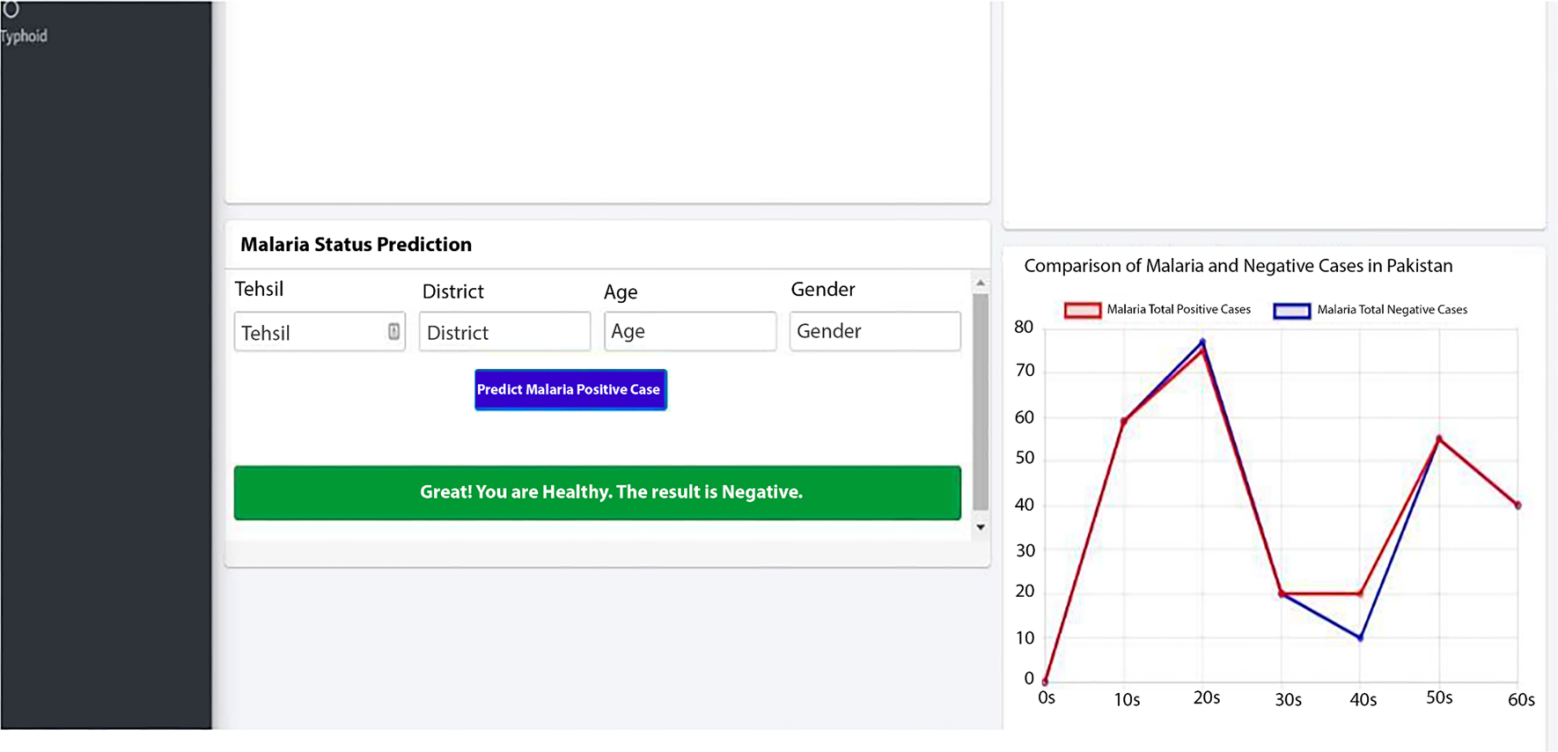
Waterborne disease dashboard snapshot 2

The findings of this study inspired us to develop an analytical dashboard. Figures 8 and 9 provide a glimpse of the analytical dashboard for our current study, which may be seen in Fig. 8. The suggested system will provide the health department with various functions. (1) The malaria module considers the patient's medical history to make a positive diagnosis. (2) It illustrates the most affected locations. (3) It illustrates the disparity in the number of positive cases found in men and women in Pakistan. (4) The typhoid module provides reliable predictions of positive cases in many places. In the end, it pinpointed the locations that were most severely affected.

### Deployment

The experimental findings of poor water quality in Pakistan show positive waterborne diseases spreading around the country. Multiple ML models were also implemented on two datasets, malaria, and typhoid patients, to accurately predict and diagnose the waterborne diseases. Thus, the learned weights of all those datasets have been employed to install the model. One of the major aims of constructing a model that accurately predicts results and is robust to changes in future knowledge. DM processes may be replicated across all businesses after the deployment phase of a new method has been completed. The useful information retrieved from the data must be sorted and presented so that anybody using the generated ML model may employ it.

The RF method delivers the most effective outcome on both datasets; therefore, it is used for training purposes. Additionally, deployment decisions likely improve the acceptability of DM technology. The methodology allowed end-users, not data miners, to participate in scenario simulation activities of the complicated system. As previously, the installation was done manually by utilizing conventional means. Deploying the model using Python is also difficult because the implementation is complicated without the API. If the deployment is robust or costly, the companies may be forced to revert to outdated models. Therefore, Python, flask, and admin LTE technologies coupled with Microsoft Azure ML Studio were used to actualize the model for waterborne illness prediction. This may deploy the model and automatically set up the model to function with Azure load-balancing technology.

## Conclusions

Diseases that are spread by water are very common in Pakistan and spread to different places because of poor hygiene. In this study, we diagnosed waterborne diseases with the help of ML algorithms. Then, real-world data is used to evaluate various ML models to identify the most suitable one for predicting positive cases of waterborne diseases with greater precision and by identifying the most notable characteristics that play a crucial role in predicting positive cases of waterborne illnesses.

The current study dataset of waterborne disease (typhoid and malaria) contained 22,916 malaria patient records and 68,624 patient records of typhoid. This study employed a set of explainable supervised ML techniques to predict waterborne disease-positive cases. The results show that RF predicts positive cases in both malaria and typhoid with high accuracy (60% for the malaria dataset and 77% for the typhoid data) compared to other classifiers. Furthermore, the results show that age, history, and test results play an important role in predicting waterborne disease-positive cases compared to other input features. Children and the elderly were the most susceptible groups since they comprised most hospitalized patients with waterborne illnesses.

*The current study has limitations* such as a lack of information on waterborne diseases and patient symptoms**.** In the future, we will use social media surveys to get other patient symptoms or input features related to waterborne disease patients. Also, ensemble methods or hyperparameter techniques will help improve the accuracy of RF models even more. Then, we will keep improving our proposed analytical dashboard and add more features. This proposed dashboard will help the hospital and government health department to make quick decisions about waterborne diseases in Pakistan. For future work, it is intended to evaluate the results of the current study to similar findings in the literature based on vastly distinct data sets.

## Data Availability

The current study data are extracted from Ayub hospital, Abbottabad, Pakistan, for research purposes. The current study data are publicly available online 7 February 2022; https://doi.org/10.7910/DVN/WBHUV5 (typhoid) 7 February 2022) for research purposes. Ethical clearance was granted by the Ayub hospital, Abbottabad, Pakistan. No participants’ personal information (e.g., name or address) was included in this study.
